# Optimization and Stability Testing of Four Commercially Available Dried Blood Spot Devices for Estimating Measles and Rubella IgG Antibodies

**DOI:** 10.1128/mSphere.00490-21

**Published:** 2021-07-14

**Authors:** Ojas Kaduskar, Vaishali Bhatt, Christine Prosperi, Kyla Hayford, Alvira Z. Hasan, Gururaj Rao Deshpande, Bipin Tilekar, Jeromie Wesley Vivian Thangaraj, Muthusamy Santhosh Kumar, Nivedita Gupta, Manoj V. Murhekar, William J. Moss, Sanjay M. Mehendale, Lucky Sangal, Gajanan Sapkal

**Affiliations:** a Diagnostic Virology Group, ICMR-National Institute of Virology, Pune, Maharashtra, India; b Department of International Health, International Vaccine Access Center, Johns Hopkins Bloomberg School of Public Health, Baltimore, Maryland, USA; c Indian Council of Medical Research (ICMR)-National Institute of Epidemiology, Chennai, India; d Division of Epidemiology & Communicable Diseases, Indian Council of Medical Research, New Delhi, India; e Department of Epidemiology, Bloomberg School of Public Health, Johns Hopkins University, Baltimore, Maryland, USA; f W. Harry Feinstone Department of Molecular Microbiology and Immunology, Bloomberg School of Public Health, Johns Hopkins University, Baltimore, Maryland, USA; g World Health Organization, Southeast Asia Region Office, New Delhi, India; University of Maryland School of Medicine

**Keywords:** dried blood spot, ELISA, HemaSpot, elution buffer, elution protocol, laboratory and field, measles, optimization, quantitative IgG titers, rubella, stability, temperature and humidity

## Abstract

Blood collection using dried blood spots (DBS) provides an easier alternative to venipuncture for sample collection, transport, and storage but requires additional processing that can cause variability in results. Whole-blood samples spotted on four DBS devices and respective paired serum samples were tested for antimeasles and antirubella IgG antibody concentrations by enzyme immunoassay. Elution protocols for DBS devices were optimized for comparability relative to serum samples using 12 adult volunteers. Stability of DBS collected on HemaSpot HF was assessed under various temperature conditions (+4, 22 to 25, and 45°C) at six time points (0, 7, 15, 30, 60, and 90 days) in a controlled laboratory setting using six adult volunteers. Devices were shipped and stored for 30 days at four settings with variable temperature and humidity conditions to assess the impact on antibody concentrations. Three DBS devices demonstrated comparable antibody concentrations with paired sera following optimization. Antibodies recovered from DBS were stable for at least 90 days at 4°C and for 30 days at ambient temperature (22 to 25°C) using the HemaSpot HF device. A drastic decline in antibody concentrations was observed at 45°C, resulting in quantitative and qualitative discrepancies by day 7. HemaSpot HF devices shipped to field sites and stored at ambient temperature and humidity resulted in quantitative, but not qualitative, variability. Measurement of antimeasles and antirubella IgG antibodies with DBS devices is an accurate alternative to testing serum, provided elution protocols are optimized. Stability of HemaSpot HF devices at ambient temperature enables broader use in surveys when serum processing and cold storage are not feasible.

**IMPORTANCE** Dried blood spot (DBS) collection offers various advantages over conventional methods of blood collection, especially when collecting and transporting samples for a serosurvey. Yet use of DBS requires additional processing steps in the laboratory that can add to variability in results. We optimized a protocol to elute IgG antibodies against measles and rubella viruses in four DBS devices, demonstrating high concordance with paired venous sera for most devices. Extensive stability studies with various temperature and storage conditions in the laboratory and in the field were conducted using HemaSpot HF DBS devices prior to its use in one of the largest community-based measles and rubella serological surveys in the world.

## INTRODUCTION

Measles and rubella are two of the most globally important vaccine preventable diseases (VPD). They are among the most infectious human diseases that can cause serious illness, lifelong complications, and death, especially among young children, despite the availability of a safe and effective vaccine. In 2012, the Measles and Rubella (MR) Initiative launched a Global Measles and Rubella Strategic Plan, 2012−2020 as a guiding document to eliminate measles and rubella (1). Through a combination of innovation, resources, and political will, India is expected to achieve measles and rubella elimination by 2023 (2).

Measuring antibodies to measles and rubella viruses in samples collected from serosurveys can play an important role in estimating population immunity and guiding immunization activities, particularly as countries move toward elimination efforts (3). Blood sample collection through venipuncture in large-scale serosurveys is often challenging or not feasible because it is an invasive technique, requires trained phlebotomists, and requires sample maintenance and transport in reverse cold chain conditions. Fingerprick collection using dried blood spots (DBS) eases these challenges with sample collection, transport, and storage and can serve as an alternative choice in research settings (4). DBS specimens, once dried and stored appropriately, are reportedly stable for months to years at ambient temperatures or under refrigeration, but stability depends on the type of DBS collection device and analyte of interest (5–9). Additionally, many studies have reported similar recovery of measles and rubella IgG antibodies on enzyme immunoassay from samples obtained by DBS and conventional venipuncture (10–13).

Whatman 903 protein saver cards are the most commonly used DBS device for measuring specific antibody concentrations (12, 14). However, there are operational challenges with the Whatman 903 card such as risk of contamination while drying and variable volume of blood spot due to under- or oversaturation of filter paper (5, 6). TropBio filter paper is another DBS device, requiring less volume per spot and easier excision of spots than Whatman 903 cards. HemaSpot HF and SE devices integrate collection and storage with an built-in desiccant and serum separation (HemaSpot SE only). We sought to compare measles and rubella IgG antibody recovery between serum samples and four DBS devices (Whatman 903, HemaSpot HF, HemaSpot SE, and TropBio) and then evaluated the effects of temperature, humidity, and storage duration on antibody concentrations using the HemaSpot HF device in controlled laboratory experiments and in real world field settings.

## RESULTS

### Optimization and comparison of DBS devices.

**FIG 1:**
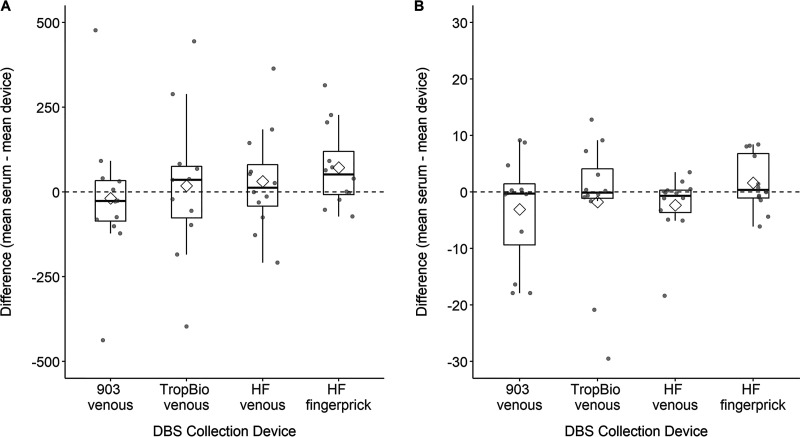
Box plot showing absolute differences in measles (A) and rubella (B) antibody concentration by DBS collection device. The DBS collection devices were Whatman 903, HemaSpot HF, HemaSpot SE, and TropBio. Twelve participants were included in the experiment. Triplicate samples were run on three separate days for a total of nine results per participant for serum sample and for each device. Mean antibody concentrations were calculated for each participant across the replicates for each device or serum sample. The plot depicts the distribution of absolute differences between the mean serum value and mean DBS device values across the 12 participants (gray circles overlaid on boxplot). The diamond represents the group mean, and the horizontal line inside the box is the median.

In the first series of experiments, DBS elution protocol and respective DBS device dilutions were optimized so that a head-to-head comparison could be done for selection of a single device for stability studies. Elution buffer (EB) was 0.1 M phosphate-buffered saline (PBS) with 0.1% Tween 20 without additional additives (see Text S1 in the supplemental material). Whatman 903 and TropBio cards gave comparable results with respective sera at 1:8 dilution with 50 μl EB, and HemaSpot HF gave optimal results at 1:8 dilution with 100 μl EB (Text S1). Three DBS devices gave qualitatively concordant results compared to serum following optimization of elution procedures (see Text S1 for detailed results). Mean antibody concentrations of the nine replicates per participant were calculated for the Whatman 903, TropBio, and HemaSpot HF with venous blood and HemaSpot HF with fingerprick blood. The median coefficients of variation were similar across devices, ranging from 8.8% to 10.5%. Compared to paired sera, there was no significant difference in mean antibody concentrations for any device compared to serum (Fig. 1; see also Table S3 in the supplemental material). Antibody concentrations obtained from fingerprick blood samples by HemaSpot HF were slightly lower than the corresponding serum samples for both measles and rubella (mean difference, sera minus HemaSpot HF fingerprick, 72.2 milli international units [mIU/ml] [*P* = 0.06] for measles and 1.6 IU/ml [*P* = 0.28] for rubella). From the perspective of the laboratory personnel collecting and processing samples, the HemaSpot HF device was favored with regard to ease of specimen collection, storage, transport, and elution (Table 1) and was therefore selected for further stability evaluation and for use in the field-based serosurveys (15).

**TABLE 1:** Summary of four dried blood spot (DBS) collection devices selected for initial evaluation

Characteristic	Whatman 903 Protein Saver	HemaSpot HF	HemaSpot SE	TropBio
Device description	Folded card with five half-inch circles printed on filter paper	Self-contained device with wedge-shaped filter paper, built-in desiccant, and a plastic cover	Self-contained device with spiral shape filter paper to separate serum, built-in desiccant, and a plastic cover	Circular card with six protrusions of filter paper, 4 cards packed in 1 sheet
Units (e.g., spots) and whole blood volume per device	5 spots∼375 μl/device∼75 μl/spot^*a*^	8 wedges∼150 μl/device^*b*^∼18.7 μl/wedge	Blood components separate out onto four sections in spiral form (whole blood, plasma, serum); no. of punched spots depend on blood component of interest∼150 μl/device^*c*^	6 protrusions∼60 μl/device∼10 μl/protrusion (23)
Collection process	Fill circles independently; potential for blood overlay	Blood flows through a center hole in device to fill all wedges simultaneously; excess blood flows to outer membrane	Blood flows through a center hole in device to spread out through filter paper	Fill protrusions independently; potential for blood overlay
Serum separation	No	No	Yes	No
Integrated collection and storage	No. Card dried >4 h in clean dry space before storage in self-sealing bag with desiccants	Yes. Device shut closed for storage soon after sample collection; built-in desiccant^*d*^	Yes. Device shut closed for storage soon after sample collection; built-in desiccant^*d*^	No. Card dried >4 h in clean dry space before storage in self-sealing bag with desiccants^*e*^
Potential for contamination with other samples	Possible. Spots are covered by a thin flap and require extended drying with flap open	Less likely. Device closed immediately after sample collection	Less likely. Device closed immediately after sample collection	Possible. Spots protrude out from center with no barrier and requires extended drying
Excision device	Punching device cleaned with alcohol between participants	Scissors and forceps for detaching wedges cleaned with alcohol between participants	Punching device cleaned with alcohol between participants	Scissors and forceps for detaching protrusion cleaned with alcohol between participants
Image^*f*^	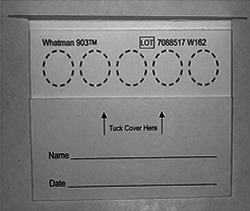	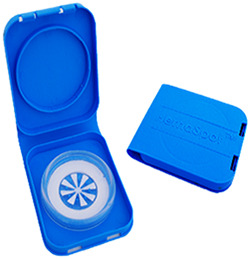	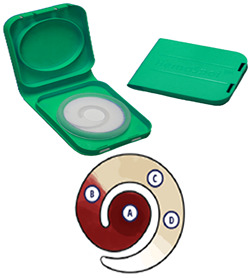	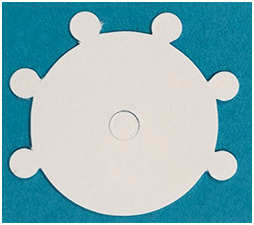
Strengths	Established card used in studies and many newborn screening programs.	Integrated collection device facilitates ease of collection.	Integrated collection device facilitates ease of collection.	Does not require punching, reduces variability in blood volume per protrusion.
	Existing evidence on comparison between card and serum samples.	Self-contained device and built-in desiccant reduce drying steps and protects from humidity.	Built-in serum separation.	Requires very small blood volume per protrusions which are independently filled.
	Requires small blood volume per spot, independently filled	Devices include barcode for sample identification.	Self-contained device and built-in desiccant reduce drying steps and protects from humidity.	
			Devices include barcode for sample identification.	
Weaknesses	Requires more extensive drying procedures.	Limited data on sensitivity and stability.	Limited data on sensitivity and stability.	Requires more extensive drying procedures.
	Elevated risk of contamination during drying or punching.	Device to be used within 30 min after opening.	Device to be used within 30 min after opening.	Elevated risk of contamination during drying.
	Potential for variability in volume between punches.	Elevated risk of undersaturation due to larger volume requirement and nonindependence of filling.	Four sections of the device are not clearly defined and concentration in each section dependent on the volume of blood collected.	

a Merck. Whatman protein saver cards 903 protein saver card (US) (package of 100 each; Sigma-Aldrich).

b Laboratory observed volume required to fully saturate device and all wedges.

c Volume per spot punched from HemaSpot SE device could not be estimated due to diffusive nature of the serum separation paper (24; also Protein Extraction Methods from HemaSpot HF or SE Devices Standard Curve Preparation).

d HemaSpot devices may be placed into a self-sealing bag with a desiccant to further prevent exposure to humidity, although this is not required (4).

e For this study, TropBio cards were packed in petri plates with desiccants out of an abundance of caution to avoid damage to the protrusion.

f HemaSpot images from manufacturer’s website (HemaSpot HF; Spot On Sciences [https://www.spotonsciences.com/hemaspot-hf]; HemaSpot SE; Spot On Sciences [https://www.spotonsciences.com/hemaspot-se]).

10.1128/mSphere.00490-21.2 TEXT S1 Optimization of dried blood spot elution procedures. Text describing the experiments conducted to optimize dried blood spot elution procedures prior to the stability experiments. Download Text S1, DOCX file, 0.2 MB. Copyright © 2021 Kaduskar et al. 2021 Kaduskar et al. https://creativecommons.org/licenses/by/4.0/ This content is distributed under the terms of the Creative Commons Attribution 4.0 International license.

10.1128/mSphere.00490-21.4 TABLE S3 Difference between means of antibody concentrations obtained from paired DBS device and serum samples. For each of 12 individuals and each sample type (sera, DBS 903, DBS HemaSpot HF fingerprick, DBS HemaSpot HF venous, DBS TropBio), the mean value was calculated across the nine replicates. The difference (or relative difference) between sample types was calculated using the mean for each individual and then summarized across all 12 individuals. Abbreviation: SD, standard deviation. Download Table S3, DOCX file, 0.01 MB. Copyright © 2021 Kaduskar et al. 2021 Kaduskar et al. https://creativecommons.org/licenses/by/4.0/ This content is distributed under the terms of the Creative Commons Attribution 4.0 International license.

### Stability experiments for HemaSpot HF devices.

**FIG 2:**
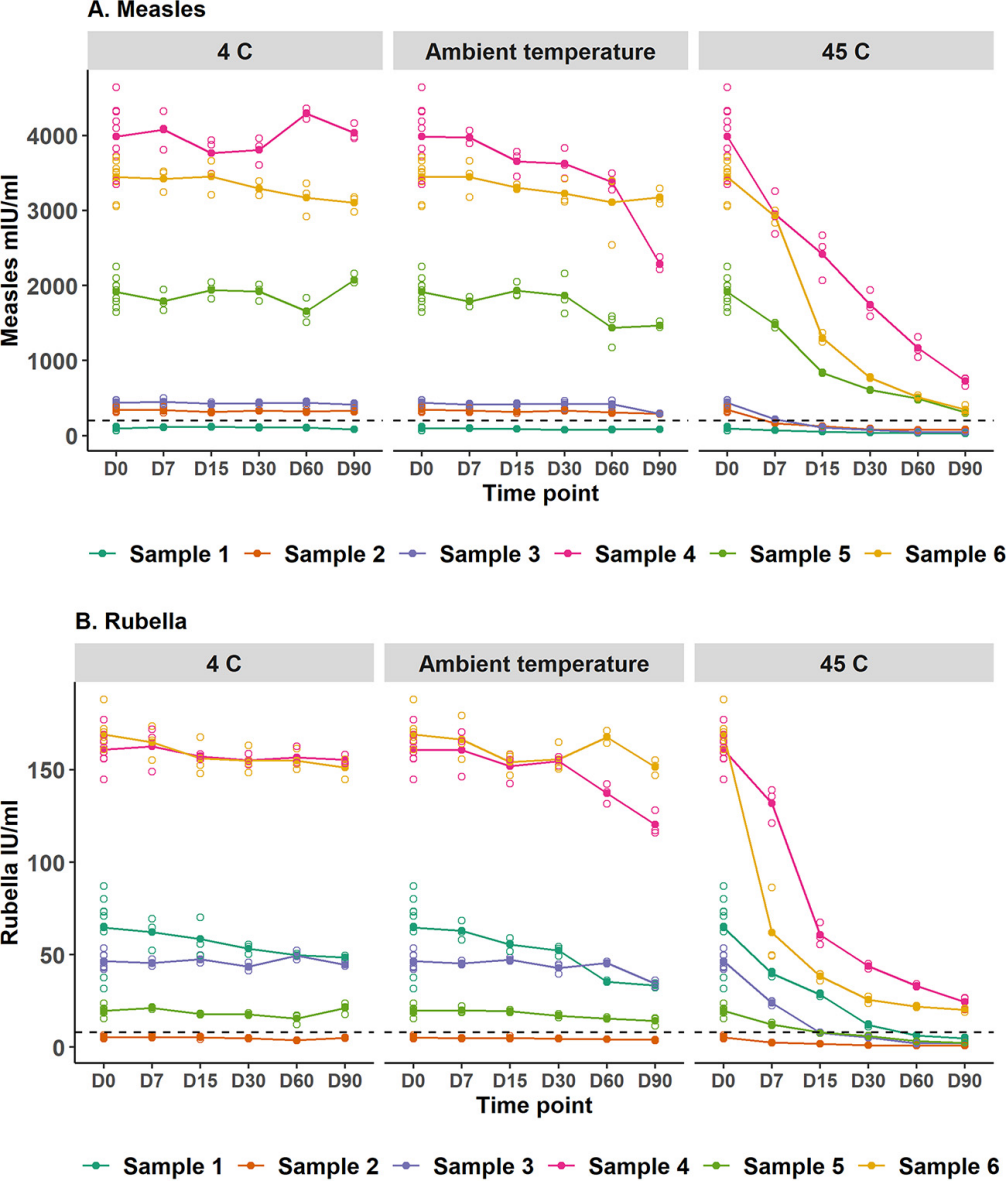
Effect of storage duration and temperature on measles (A) and rubella (B) IgG antibodies obtained from HemaSpot HF devices stored at the central laboratory. The ambient temperature in the laboratory was an average temperature of 24.8°C (range, 24 to 26.7) and average humidity of 73.8% (range 63 to 80%). The average relative humidity (RH) at 4°C was 92.1% (maximum RH, 100%). The average RH at 45°C was 16.7% (maximum RH, 20.1%). Day 0 is the same in all three temperature graphs, reflecting day 0 at ambient temperature where nine replicates were available for each participant. For all other time points, the samples were run in triplicates. Solid circles represent the average of the replicates, and open circles represent the values for individual replicates. The dashed black line indicates 200 mIU/ml for measles and 8 IU/ml for rubella (threshold between negative and equivocal; values above the dashed black line were categorized as positive in this analysis).

Antibody stability of DBS collected using the HemaSpot HF device varied by storage temperature. At 4°C, measles IgG concentration remained at least 95% of baseline levels through 90 days of storage (Fig. 2 and Table S4). At ambient temperature, measles IgG concentrations were stable for 30 days and dropped below 90% of baseline at days 60 and 90. At 45°C, measles IgG concentrations declined immediately after day 0, dropping below 70% of baseline by day 7 and below 30% of baseline by day 30 (Table S4). Similar findings were observed for rubella. No qualitative change in measles or rubella IgG results was observed at 4°C or ambient temperature at any time point.

10.1128/mSphere.00490-21.5 TABLE S4 IgG antibody concentration at each temperature and time point relative to baseline. The HemaSpot HF devices were stored at various temperatures and durations. The baseline is the day 0 HemaSpot HF result. Relative humidity (RH) in each laboratory setting: at ambient temperature, average RH of 73.8% and max RH of 80%; at 4°C, average RH of 92.1% and max RH of 100%; at 45°C, average RH of 16.7% and max RH of 20.1%. Ambient temperature (average temperature 24.8°C (range, 24 to 26.7°C). Download Table S4, DOCX file, 0.01 MB. Copyright © 2021 Kaduskar et al. 2021 Kaduskar et al. https://creativecommons.org/licenses/by/4.0/ This content is distributed under the terms of the Creative Commons Attribution 4.0 International license.

**FIG 3:**
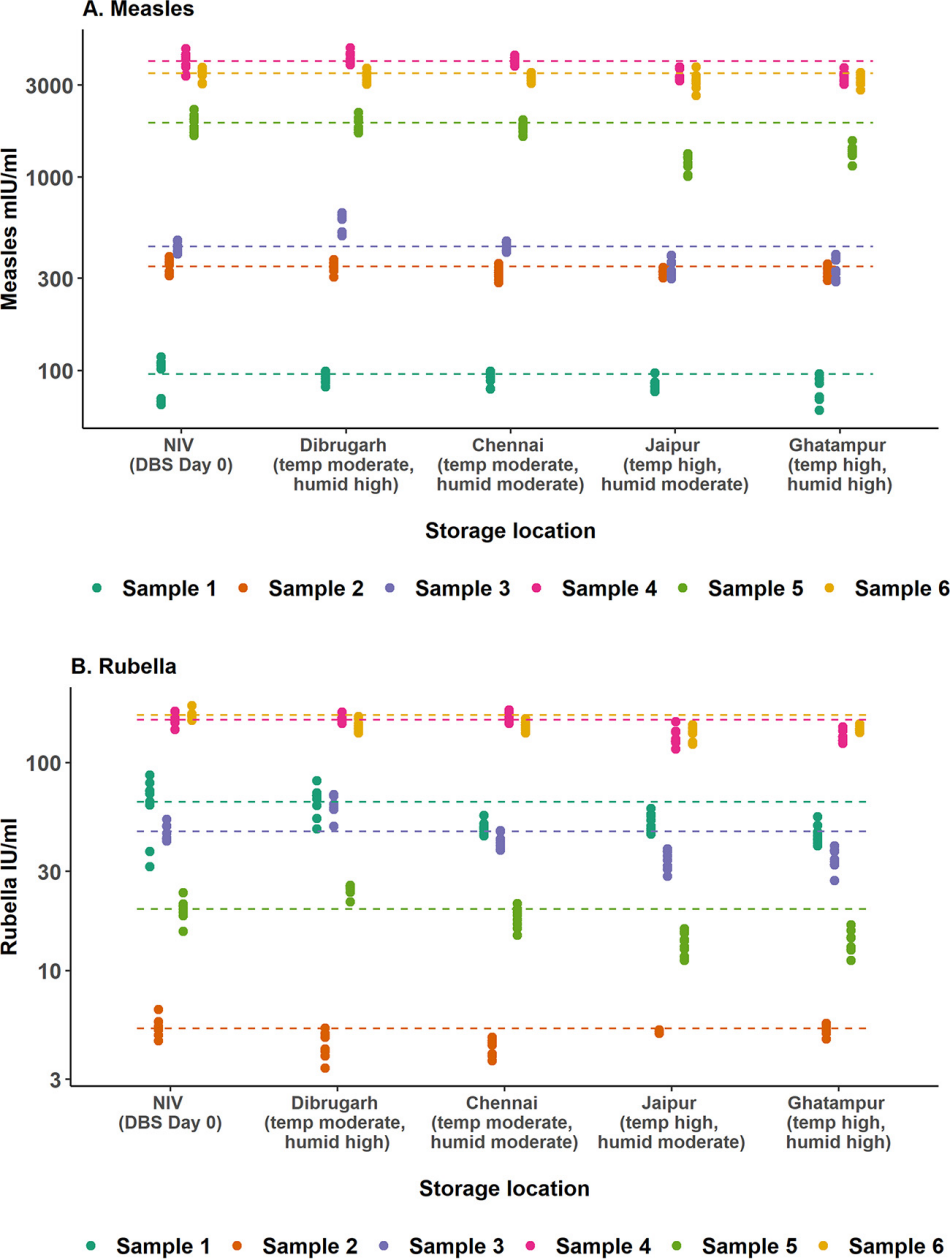
Measles (A) and rubella (B) IgG antibody concentrations obtained from HemaSpot HF devices stored for 30 days at sites with various temperature and humidity conditions. Each dot represents the value for one of the nine replicates for each storage site and participant. The dashed horizontal line represents the mean antibody concentration from the DBS day 0 (D0) samples. Temperature and humidity conditions reflect when the experiment was conducted (July 2019).

We extended the stability evaluation to field-based settings where temperature and humidity were not controlled and varied throughout each day and between days. For four of six samples, significantly lower measles IgG concentration were observed after 30 days compared to day 0 baseline values at the Jaipur and Ghatampur sites in India, after correcting for multiple comparisons (Fig. 3 and Table 2). Similar findings were observed for rubella antibodies, where concentrations were lower following 30 days for four of six samples in these sites. Both sites reflect settings with high temperature and moderate-to-high humidity (Table 3 and Fig. S1). There were no differences in qualitative results at the four sites compared to concentrations at day 0.

**TABLE 2:** Difference in mean IgG antibody concentration obtained from dried blood spots, comparing baseline (day 0 samples stored at central laboratory) with samples stored for 30 days at sites

Antibody and ID^*a*^	Day 0 mean^*b*^	Dibrugarh		Chennai		Jaipur		Ghatampur	
		Mean difference^*c*^	*P* value^*d*^	Mean difference	*P* value	Mean difference	*P* value	Mean difference	*P* value
Measles									
1	95.7	−3.8	0.59	−3.7	0.61	−10.8	0.14	−13.6	0.10
2	345.8	−2.0	0.87	−26.3	0.06	−24.5	0.04	−24.5	0.07
3	437.5	153.1	**<0.001**	2.9	0.80	−95.0	**<0.001**	−92.2	**<0.001**
4	3,986.9	182.8	0.31	40.3	0.80	−647.6	**0.001**	−641.5	**0.001**
5	1,917.0	−62.3	0.47	−123.9	0.12	−715.18	**<0.001**	−560.6	**<0.001**
6	3,445.9	−104.6	0.33	−150.8	0.12	−297.3	0.03	−229.6	0.05
Rubella									
1	64.6	1.3	0.86	−16.0	0.02	−13.3	0.06	−19.2	0.01
2	5.3	−0.7	0.03	−0.9	**0.001**	−0.2	0.46	−0.0	0.92
3	46.7	16.4	**<0.001**	−4.6	0.02	−11.9	**<0.001**	−12	**<0.001**
4	161.0	4.2	0.27	5.0	0.23	−28.5	**<0.001**	−26.7	**<0.001**
5	19.8	5.0	**<0.001**	−2.2	0.05	−6.1	**<0.001**	−6.1	**<0.001**
6	169.1	−18.4	**<0.001**	−18.1	**<0.001**	−31.3	**<0.001**	−23.8	**<0.001**

a ID, identifier.

b D0, day 0.

c Mean difference calculated as day 30 site value minus day 0 value calculated as the mean across the nine replicates for each individual.

d *P* values obtained from one-way analysis of variance test. Boldface values show *P* < 0.002 (Bonferroni-corrected *P* value accounting for 24 pairwise comparisons).

**TABLE 3:** Temperature and humidity conditions at storage sites

Characteristic	Dibrugarh	Chennai	Jaipur	Ghatampur
Temperature				
Mean (SD) (sites)^*a*^	28.6 (1.7)	30.2 (1.1)	30.0 (1.3)	32.5 (1.3)
Mean (SD) (weather station)^*b*^	27.7 (2.3)	31.6 (1.6)	30.5 (2.5)	29.7 (2.1)
Humidity, mean (SD) (sites)^*a*^	95.4 (5.0)	60.0 (6.3)	61.7 (10.8)	69.5 (5.0)

a Temperature and humidity reflect the mean of the afternoon measurements obtained by staff. Temperature and humidity conditions of the four sites (July 2019) are as follows: Dibrugarh, moderate temperature, high humidity; Chennai, moderate temperature, moderate humidity; Jaipur, high temperature, moderate humidity; Ghatampur, high temperature, higher humidity.

b Temperature from weather station reflect outside temperature obtained from Global Historical Climatology Network - Daily (GHCN-Daily) data set (21, 22). For Ghatampur, the nearest weather station with available data is Lucknow (approximately 96 km northeast).

10.1128/mSphere.00490-21.6 FIG S1 Temperature (A) and humidity (B) measurements at four DBS storage locations. Temperature and humidity were measured twice daily (morning and afternoon/evening). Each measurement is displayed in the graph. The red line in the temperature graphs reflects outside temperature from weather stations obtained from Global Historical Climatology Network - Daily (GHCN-Daily) data set (21, 22). For Ghatampur, the nearest weather station with available data is Lucknow (approximately 96 kilometers northeast). Download FIG S1, TIF file, 2 MB. Copyright © 2021 Kaduskar et al. 2021 Kaduskar et al. https://creativecommons.org/licenses/by/4.0/ This content is distributed under the terms of the Creative Commons Attribution 4.0 International license.

## DISCUSSION

Here, we reported the performance of multiple DBS collection devices compared to sera for quantifying measles and rubella IgG antibodies after extensive elution optimization experiments. Based on findings from the optimization experiments and the experience of the laboratory personnel using the four different DBS devices, the HemaSpot HF device was selected for further DBS stability assessment under different temperature and humidity conditions in the controlled laboratory settings and real world field conditions.

Our study reports extensive optimization experiments for four types of DBS collection devices with various elution buffer volumes and eluate dilution factors to accurately quantify both measles and rubella IgG antibodies (see Text S1 in the supplemental material for detailed methods and analyses). Optimization of elution conditions is a key step prior to initiating testing of DBS samples, since factors such as hematocrit, blood volume per spot, and filter paper characteristics contribute to different extraction yields of a DBS sample (6), which may lead to false-positive or false-negative results compared to testing serum. Further, it is important to determine the blood volume in one unit (spot, protrusion, or wedge) of each DBS device (16) (Table 1 and Text S1) and verify that the DBS extraction, elution, and assay generate accurate results compared to testing serum or plasma.

We observed stability of antimeasles and antirubella antibodies obtained from HemaSpot HF devices stored at 4°C for up to 90 days. Minor degradation in antibody concentration was observed when stored at ambient temperatures (22 to 25°C) for more than 30 days, but it did not affect qualitative results. Our results suggest that if storage is anticipated beyond 30 days, then the spotted HemaSpot HF devices should be transferred to 4°C. Although we did not control for humidity in these experiments, we measured humidity in the different settings and limited exposure to humidity by sealing the devices inside a bag with a desiccant. Another study that assessed HIV antibody stability on HemaSpot HF devices observed no degradation at room temperature with less than 40% humidity or 37°C at 95% humidity through 180 days of storage when sealed and stored with desiccants. Antibody degradation was observed after 180 days at 37°C when the HemaSpot HF device was not stored in sealed bags, suggesting temperature and humidity were important for antibody stability (4). For devices stored at 45°C, we observed substantial antibody deterioration at the first time point (7 days), indicating HemaSpot HF devices should not be stored at high temperatures for any period of time. Manak et al. (4) also observed degradation of HIV antibodies at 45°C after day 30 and day 180 for HemaSpot HF devices in open and sealed storage bags, respectively.

Studies conducted only in laboratory settings do not replicate the variability in temperature and humidity within and between days as observed in field settings. To address this, six DBS samples collected with HemaSpot HF devices were shipped and stored in four different settings in India that varied in temperature and humidity. Comparatively lower antibody concentrations were observed relative to baseline following 30 days of storage in Jaipur and Ghatampur (mean temperature. 30 to 33°C; mean humidity, 62 to 70%), but there were no qualitative changes observed. These findings provide additional evidence that DBS samples collected using HemaSpot HF devices can be safely stored and transported at ambient temperatures for at least 30 days; however, we recommend any longer duration of storage be done at 4°C. Despite high humidity in the Dibrugarh setting, there was limited quantitative impact on measles antibody recovery for those devices. We hypothesize the similar antibody concentrations relative to baseline may have been due to the devices having limited exposure to the various conditions and high humidity given that the filter paper was sealed in a plastic device inside a self-sealing bag with a separate desiccant.

On assessing three DBS collection devices (Whatman 903, HemaSpot HF, and TropBio) for their reproducibility and accuracy, we found that all devices were qualitatively concordant with its corresponding serum specimens for measles and rubella IgG antibodies. Quantitatively, the mean measles and rubella IgG antibody concentrations recovered from the DBS devices spotted with venous blood were similar to the corresponding serum samples. This is consistent with prior research where DBS had >90% antibody recovery (11, 12, 17–19). The HemaSpot HF device spotted directly from fingerprick blood produced antibody concentrations slightly lower than those found with serum samples but with no difference in qualitative results. This observation could be attributed to the fact that the concentration of antibodies in fingerprick blood is lower than venous blood (20). The HemaSpot SE device showed considerable variability in results compared to the corresponding serum sample, and concentration is dependent on where the filter paper is punched. After the initial optimization experiments, this device was excluded from future experiments despite attractive features such as serum separation.

We also compared the four DBS collection devices based on their characteristics and ease of use (Table 1). TropBio and Whatman 903 cards are often preferred given their relatively low cost. However, there is the potential for accidental splatter, smearing, and either over- or undersaturation of filter paper (4), although these issues may be minimized by proper training. Compared to other devices, sample collection was theoretically and practically easier with the HemaSpot HF device where the blood drops on the plastic covering and falls through the center hole onto the filter paper. Any excess blood flows out of the wedge to the outer filter ring, resulting in a uniform volume of sample in the eight wedges with less likelihood of oversaturation. Approximately 150 μl of blood is required to fully saturate all wedges of the HemaSpot HF device, which may be difficult to collect, especially from young children. Undersaturation and loss of sample are significant risks for the HemaSpot HF device because all wedges are affected when insufficient blood is applied. Whatman 903 or TropBio cards are less at risk for undersaturation because spots are filled independently. The HemaSpot HF device is a self-contained unit with built-in desiccants to ensure efficient drying of the sample, simplifying the drying, storage, and shipment processes. In contrast, both the Whatman and TropBio cards need to be air dried for at least 4 h and then individually packed in self-sealing bags for storage and shipment. DBS elution procedures are simpler with HemaSpot HF device and TropBio cards, as the wedges and protrusions can be easily separated by forceps and scissors, whereas Whatman 903 and HemaSpot SE devices require punching. While HemaSpot HF devices are approximately two to three times more expensive than Whatman 903 or TropBio cards, its design characteristics may allow for easier storage, transport, and elution. Preferences from the field and laboratory staff should be considered when selecting a device.

Limitations of this work include small sample sizes, ranging from 4 to 12 participants per experiment. Although humidity was different for each storage temperature in the laboratory experiments, additional analyses changing both temperature and humidity would be valuable. Strengths include a comprehensive series of experiments optimizing multiple aspects of the elution and testing procedure and a head-to-head comparison of four devices. The additional stability studies using HemaSpot HF devices provide important findings on optimal storage temperature and duration of storage applicable to future studies implementing collection with this device.

Collection of filter paper blood spots is a promising alternative to testing plasma or serum for detection of measles and rubella IgG antibodies using commercially available Euroimmun enzyme immunoassays (EIAs). DBS samples are particularly valuable for field-based surveys to address challenges of on-site sample processing, transport, and temperature-controlled storage. However, improperly handled and stored samples may result in antibody degradation and incorrect results. Accounting for the temperature and humidity conditions in the study setting, and duration of storage at those conditions, is important when developing specimen handling procedures to ensure the quality remains intact.

## MATERIALS AND METHODS

### Specimen collection.

Four commercially available DBS collection devices were selected in the study: Whatman 903 card (catalog no. 10550021; GE Healthcare Ltd., Cardiff, United Kingdom), HemaSpot HF blood collection device (catalog no. H0001; Spot on Science, San Francisco, CA, USA), HemaSpot SE blood separation device (catalog no. H010; Spot on Science, San Francisco, CA, USA), and TropBio card (catalog no. 05-002-12; Cellabs, Sydney, Australia) (Table 1). Participants were selected for these experiments from a pool of healthy adult volunteers at the Indian Council of Medical Research (ICMR)-National Institute of Virology (NIV), Pune, India, based on known measles and rubella IgG concentrations. The pool of participants reflects a wide range of IgG levels for measles (range, 77.5 to 4,255.2 mIU/ml) and rubella (range, 0.7 to 771.3 IU/ml).

Five milliliters of blood was collected from each participant by venipuncture in a BD vacutainer serum separator tube (SST) (catalog no. 367954; Becton Dickinson and Company, Franklin Lakes, NJ, USA) and a BD vacutainer EDTA tube (catalog no. 367525; Becton Dickinson and Company, Franklin Lakes, NJ, USA). We spotted 25 μl of EDTA blood on each of the five circles of Whatman 903, 10 μl on each protrusion of TropBio card, and 150 μl in the center of the HemaSpot HF and HemaSpot SE devices. In addition, 4 or 5 drops of fingerprick blood were directly spotted on the HemaSpot HF device. Blood in SST were processed as sera, aliquoted, and stored at 4°C for a maximum of 1 week; otherwise, it was stored at −20°C until used.

Whatman 903 and TropBio cards were air dried overnight, while HemaSpot HF and SE devices were shut closed immediately after collection, as recommended by the manufacturers. TropBio cards were placed into individual petri plates out of an abundance of caution to avoid damage to protrusions while drying. All DBS devices were then stored in separate plastic bags with desiccants at 2 to 8°C until testing.

### Specimen processing and testing.

DBS samples were removed from storage and equilibrated at ambient temperature (average, 24.8°C [range, 24 to 26.7°C], relative humidity 73.8% [range, 63 to 80%]) for 20 to 30 min. One 6-mm spot from Whatman 903 card and HemaSpot SE device was punched using a hole punch device (catalog no. 70017; Janrax Global, China). One wedge for the HemaSpot HF device and one protrusion for the TropBio card were excised using scissors and forceps. One unit (i.e., spot, wedge, protrusion) was then placed in a self-standing 1.8-ml tube; the required volume of elution buffer (EB) was added, and the tube was vortexed for 15 to 30 s (see Text S1 in the supplemental material). The tubes were agitated twice in a shaker at 200 rpm for 20 min at 37°C with a 2-h resting period at 37°C between the agitation steps. DBS filter papers were discarded, tubes were centrifuged at 3,500 relative centrifugal force (RCF) for 10 min at ambient temperature, and eluate was collected.

Enzyme immunoassays (EIAs) for antimeasles IgG (catalog no. EI2610-9601G; Euroimmun, Lubeck, Germany) and antirubella IgG (catalog no. EI2590-9601G; Euroimmun, Lubeck, Germany) were used per manufacturer’s instructions for all experiments. Serum and DBS eluate samples were diluted to achieve a final sample dilution of 1:101, consistent with manufacturer instructions. Results were evaluated quantitatively by using the plate-specific standard curve developed by plotting the extinction values of four calibrators. Samples were classified as positive, negative, or equivocal, per manufacturer thresholds (see Table S1 in the supplemental material).

10.1128/mSphere.00490-21.1 TABLE S1 Euroimmun recommended interpretation of quantitative results for measles and rubella EIA testing. Categorization was done per the kit recommendations. Download Table S1, DOCX file, 0.01 MB. Copyright © 2021 Kaduskar et al. 2021 Kaduskar et al. https://creativecommons.org/licenses/by/4.0/ This content is distributed under the terms of the Creative Commons Attribution 4.0 International license.

### Optimizing DBS elution protocols.

Elution and dilution protocols were optimized for comparability of antibody concentrations across devices (Text S1). Following optimization, 12 individuals from the pool of volunteers were selected to compare antibody recovery of three DBS devices (Whatman 903, HemaSpot HF, and TropBio cards). HemaSpot SE was excluded following the initial optimization phase due to observed variability with serum specimens in the optimization experiments and challenges in determining the appropriate location (section A, B, C, or D) to punch given the serum separation design. Volunteers were selected to represent a broad range of measles IgG concentrations: one negative, three low positives (antimeasles IgG concentrations in a range of 275 to 550 mIU/ml), three moderate positives (551 to 2,500 mIU/ml), and five high positives (>2,500 mIU/ml). The antirubella IgG concentrations of these 12 subjects were 2 negatives, 4 low positives (11 to 50 IU/ml), 4 moderate positives (51 to 100 IU/ml), and 2 high positives (>100 IU/ml). The devices were spotted with venous blood using a pipettor as described above, and an additional HemaSpot HF device was spotted with fingerprick blood. Paired serum specimens were run on the same plates. All samples were run in triplicate on 3 days to assess reproducibility and precision.

### Stability experiments for HemaSpot HF devices.

Stability of measles and rubella IgG antibodies recovered from HemaSpot HF devices was evaluated under various temperatures, humidity, and durations in controlled laboratory conditions and with devices stored in field settings (Table S2). Venous blood samples were collected from six volunteers at ICMR-NIV, Pune, India, and 150 μl was spotted on HemaSpot HF devices using micropipettes. The devices were stored in the laboratory in sealed bags with desiccants at three different temperatures: 4°C (average relative humidity [RH], 92.1%; maximum RH, 100%), ambient temperature (in non-air-conditioned room, 22 to 25°C; average RH, 73.8%; maximum RH, 80.0%), and 45°C (incubator without humidity control; average RH, 16.7%; maximum RH, 20.1%) and tested at six time points (days 0, 7, 15, 30, 60, and 90). To assess antibody stability under field conditions, HemaSpot HF devices for the six participants were also shipped at ambient temperature and humidity to four different sites in India in July, 2019: Chennai, Tamil Nadu, a coastal region with moderate temperature and moderate humidity; Dibrugarh, Assam, a foothill area with moderate temperature and high humidity; Ghatampur, Uttar Pradesh, with high temperature and high humidity; and Jaipur, Rajasthan, a desert topography with high temperature and moderate humidity. Temperature and humidity categorizations are based on approximate conditions in these settings for July. The devices were stored in non-air-conditioned rooms for a period of 30 days and shipped back to ICMR-NIV, Pune, India, at ambient temperature and humidity. Temperature and humidity were measured in the room where devices were stored in the morning and evening. Outside temperature measurements were obtained from weather stations located near each site (21, 22). DBS readings from day 0 spotted whole blood stored at NIV served as baseline for comparison in both the laboratory and field settings. Tests were carried out in triplicate on three separate days.

10.1128/mSphere.00490-21.3 TABLE S2 Laboratory and field-testing plan to assess antibody concentrations with HemaSpot HF device stability over various times and temperature and humidity conditions. Footnote a, Six individuals were tested in triplicate for each time point for both measles and rubella. Footnote b, Relative humidity (RH) in each laboratory setting: at ambient temperature, average RH of 73.8% and max RH 80%; at 4°C, average RH of 92.1% and max RH of 100%; at 45°C, average RH of 16.7% and max RH of 20.1%. Footnote c, baseline comparison for all other conditions (laboratory and field settings) and subsequent time points. Download Table S2, DOCX file, 0.01 MB. Copyright © 2021 Kaduskar et al. 2021 Kaduskar et al. https://creativecommons.org/licenses/by/4.0/ This content is distributed under the terms of the Creative Commons Attribution 4.0 International license.

### Statistical analysis.

Absolute difference in the IgG antibody concentration (mean serum minus mean DBS) for measles and rubella was estimated for each individual and paired *t* tests were conducted. For the laboratory stability experiments, quantitative antibody concentrations were compared as a percentage of baseline values; the mean result from each storage condition and duration combination was divided by the baseline mean (day 0 DBS specimen) for each individual and then summarized across all individuals. For the field setting stability experiments, one-way analysis of variance (ANOVA) tests were performed to compare the mean values between the storage locations, and the *P* value for significance was adjusted to account for the multiple comparisons (24 pairwise comparison; Bonferroni *P* < 0.002). Analyses were conducted using SAS version 9.4 (SAS, Cary, NC, USA) and R (version 3.4.4, Vienna, Austria).
