# Supplementary material for: Optimization and Stability Testing of Four Commercially Available Dried Blood Spot Devices for Estimating Measles and Rubella IgG Antibodies
Source: mSphere. 2021 Jul 14;6(4):e00490-21. doi: 10.1128/mSphere.00490-21 (PMC8386427; doi:10.1128/mSphere.00490-21)
Supplement: TEXT S1 [file msphere.00490-21-t0001.docx]

**Supplemental Text Se: Optimization of Dried Blood Spot Elution Procedures**

**Methods: Optimizing DBS elution protocols**

The aim of the first series of experiments was to optimize elution and enzyme immunoassay (EIA) testing procedures for DBS specimens with different collection devices (Table 2). DBS elution procedures, including composition of elution buffer (EB) and eluate dilution factor, were first optimized on Whatman 903 cards for detecting measles and rubella IgG using Euroimmun kits (Experiment 1). Using 0.1M phosphate buffered saline (PBS) with varying concentrations of Tween20 as EB, the eluate was diluted in four dilutions (1:2, 1:6, 1:8 and 1:11) using Euroimmun EIA sample diluent. The second experiment compared the effects of three additives (0.8% NH_3_ [ammonia], 5% skim milk and 10% NaN_3_ [sodium azide]) on antibody recovery [1][2][3][4][5][6]. Using the optimal EB identified in the prior experiments, we then evaluated how to adapt the elution protocol and dilution factors for the other DBS devices (Whatman 903, HemaSpot HF, HemaSpot SE, and TropBio). For each device, we compared two EB volumes (50µ and 100µl) and 4 eluate dilutions. We used 1:8, 1:11 and 1:16 dilutions with eluate obtained from 50µl EB volume and 1:6, 1:8 and 1:11 dilutions with eluate obtained using 100µl EB volume (Experiment 3). Each of these experiments was performed using specimens from four participants with paired serum specimens run simultaneously serving as the reference (Table 1). Specimens were tested in duplicate on each plate and repeated on separate days.

**Table 1: Overview of experiments conducted to optimize extraction of antibodies from DBS compared to serum**

|  | Experiment 1 | Experiment 2 | Experiment 3 | | Experiment 4^a^ |
| --- | --- | --- | --- | --- | --- |
| Aim | Optimize concentration of Tween 20 and eluate dilution | Effect of elution buffer additives | Optimize elution buffer volume and eluate dilution using alternative DBS devices | | To compare results between devices and sera |
| DBS device(s) used | 903 | 903 | 903, HF, SE, and TB | | 903, HF, and TB |
| Elution buffer | **0.1 M PBS** | 0.1 M PBS + 0.1% Tween 20 | 0.1 M PBS + 0.1% Tween 20 | | 0.1 M PBS + 0.1% Tween 20 |
| Additives | **0.1% Tween 20**  0.2% Tween 20  0.5% Tween 20 | **None**  0.8% NH_3_  5% skim milk  10% NaN_3_ | None | | None |
| Elution buffer vol. | 50 µl | 50 µl | **50 µl^b^** | **100 µl^c^** | 50 µl for 903 and TB  100 µl for HF |
| DBS eluate final dilution^d^ | 1:2  1:6  **1:8**  1:11 | 1:8 | **1:8^b^**  1:11  1:16 | 1:6  **1:8^c^**  1:10 | 1:8 |
| No. participants | 4 | 4 | 4 | | 12 |
| No. replicates | Duplicates | Duplicate on 3 separate days | Duplicate on 2 separate days | | Triplicate run on 3 separate days |

Bold text indicates selected parameters for further experiments.

a. Methods and results presented in main paper.

b. Selected optimization for Whatman 903 and TropBio cards.

c. Selected optimization for HemaSpot HF.

d. Dilution ratio: 1:2 (50 µl elute+ 50 µl Euroimmun Sample Diluent), 1:6 (16.67 µl elute+ 83.33 µl Euroimmun Sample Diluent),1:8 (12.5 µl elute+ 87.5 µl Euroimmun Sample Diluent), 1:11 (9.09 µl elute+ 90.91 µl Euroimmun Sample Diluent), 1:16 (6.25 µl elute+ 93.75 µl Euroimmun Sample Diluent).

**Results: Optimizing DBS elution protocols**

Firstly, the composition of EB containing 0.1M PBS with varying concentrations of Tween 20 and eluate dilution for EIA was optimized. EB comprising of 0.1M PBS with 0.1% Tween 20 with 1:8 eluate dilution for a Whatman 903 card specimens gave readings most comparable with its corresponding serum specimen (Figure 1).

**Figure 1: Effect of Tween 20 concentration and eluate dilution on (a) measles and (b) rubella antibody concentration (Experiment 1)**

**a. Measles**


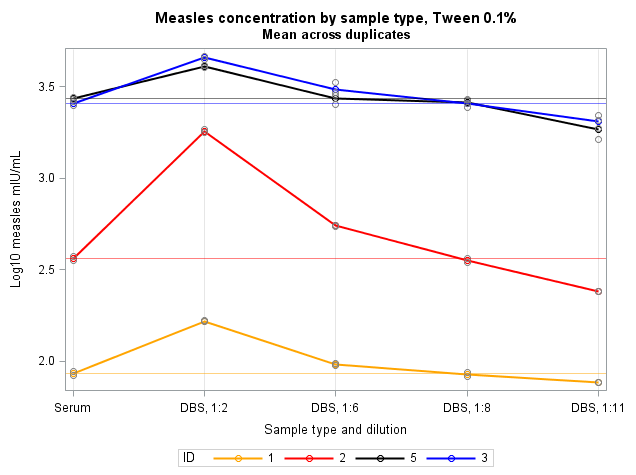


**b. Rubella**


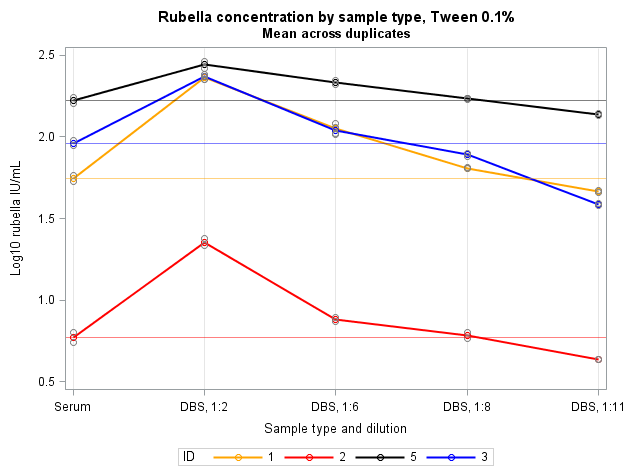


Gray markers represent observed values (4 individuals tested in duplicate for both measles and rubella), colored straight lines/markers represent mean of observed values.

None of the additives had an enhancing effect on elution capacity of the buffer (Figure 2).

**Figure 2: Effect of DBS elution buffer additives on (a) measles and (b) rubella antibody concentration (Experiment 2)**

**a. Measles**


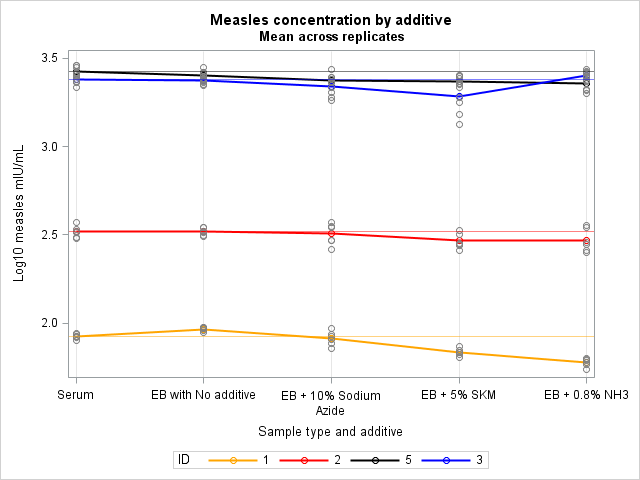


**b. Rubella**


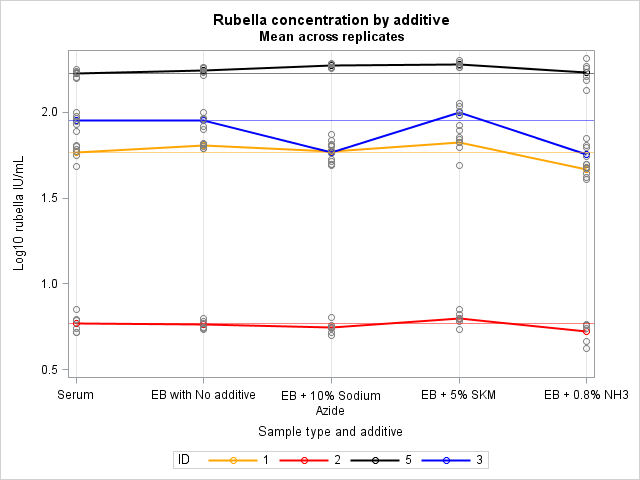


Gray markers represent observed values (4 individuals tested in duplicate for both measles and rubella), colored straight lines/markers represent mean of observed values.

Because we hypothesized different DBS devices hold different amounts of whole blood/ serum per unit (1 wedge of HemaSpot HF, 1 protrusion of TropBio and one 6 mm spot for HemaSpot SE and Whatman 903 card), we optimized the EB volume and dilution factor for each of these devices. Measles and rubella IgG concentrations from Whatman 903 and TropBio cards eluted in 50µl of EB volume with an eluate dilution of 1:8 were most comparable to the respective serum specimen (Figure 1 [903] and Table 2 [TropBio]). Measles and rubella IgG concentrations from HemaSpot HF specimens were most comparable with kit recommended serum specimen when using 50µl EB with eluate dilution 1:16 and 100µl of EB with 1:8 eluate dilution (Table 2). For further experiments we selected 100µl EB with 1:8 eluate dilution for HemaSpot HF devices as it provided us with greater volume of eluted sample which can be used for other tests or archiving.

**Table 2: Adapting the elution protocol and dilution factors for two other DBS devices: Mean difference in (A) measles and (B) rubella antibody concentration (95% CI) relative to sera sample (Experiment 3)**

**A. Measles**

|  |  | Mean difference in antibody concentration (95% CI) relative to sera sample (sera minus DBS) | |
| --- | --- | --- | --- |
| Elution Volume | Dilution Factor | HemaSpot HF | TropBio |
| 50 µL | 1:8 | -907 (-2242, 428) | 26 (-125, 176) ^a^ |
|  | 1:11 | -586 (-1525, 353) | 459 (-305, 1222) |
|  | 1:16 | -94 (-318, 130) | 803 (-518, 2124) |
| 100 µL | 1:6 | -398 (-1220, 424) | 699 (-472, 1872) |
|  | 1:8 | -89 (-440, 263)^a^ | 817 (-529, 2165) |
|  | 1:11 | 423 (-351, 1198) | 933 (-592, 2457) |

For each of 4 individuals and each sample type (sera, DBS HemaSpot HF, DBS TropBio), the mean value was calculated across the 4 replicates. The difference between sample types was calculated for each individual using the mean value then summarized across all 4 individuals.

a. indicates elution volume and dilution factor selected for subsequent experiments.

**B. Rubella**

|  |  | Mean difference in antibody concentration (95% CI) relative to sera sample (sera minus DBS) | |
| --- | --- | --- | --- |
| Elution Volume | Dilution Factor | HemaSpot HF | TropBio |
| 50 µL | 1:8 | -62 (-138, 14) | 4 (-22, 29) ^a^ |
|  | 1:11 | -38 (-92, 16) | 20 (-16, 55) |
|  | 1:16 | -7 (-29, 16) | 33 (-3, 70) |
| 100 µL | 1:6 | -34 (-73, 5) | 32 (-3, 67) |
|  | 1:8 | -2 (-20, 17)^a^ | 45 (-5, 95) |
|  | 1:11 | 22 (-1, 46) | 59 (-16, 133) |

For each of 4 individuals and each sample type (sera, DBS HemaSpot HF, DBS TropBio), the mean value was calculated across the 4 replicates. The difference between sample types was calculated for each individual using the mean value then summarized across all 4 individuals.

a. indicates elution volume and dilution factor selected for subsequent experiments.

Results obtained with the HemaSpot SE device were quantitatively varying with that of the respective sera (data not shown). Also due to the serum separation design of the HemaSpot SE, determining the appropriate location (section A, B, C or D) to punch and estimating the volume of whole blood or serum was challenging. Consequently, the device was not included in subsequent experiments.

**Interpretation: Optimizing DBS elution protocols**

Blood Volume and Elution Efficiency

When spotting DBS with whole blood it is critical to know the exact volume of blood/serum in one unit (spot, protrusion or wedge) of that device for developing and executing a reliable test[7]. Assuming blood is absorbed homogeneously on the DBS device, there is a direct correlation between spot size and quantity of whole blood[8]. The volume of blood absorbed correlates well with the density of the filter paper. The type and brand of filter paper influences the interaction between spot area and blood volume[9]. Since each DBS device hold different volume of blood[10][11][12], elution efficiency was estimated for each device depending on their final eluate dilution. Here, the elution efficiency refers to the dilution of DBS eluate obtained from each device that gives equivalent antibody concentration to serum.

One circle of Whatman 903 protein saver card is 12.7mm in diameter and can absorb approximately 75µl of whole blood[10]. Hence, a 6mm DBS spot contains about 16µl of whole blood and approximately 8 µl of serum. As we optimized 1:8 DBS eluate dilution in 50µl elution buffer for Whatman 903 protein saver cards, we get a final serum dilution of 1:50 using the DBS device as compared to recommended dilution of 1:101 for actual serum samples using Euroimmun IgG EIAs for measles and rubella. Similarly for TropBio DBS device, which is estimated to contain approximately 10µl of whole blood per protrusion, the final serum dilution is 1:80[12]. According to Hemaspot HF manual, the device absorbs approximately 80µl whole blood for entire device[11]. Available literature describes the capacity of the device ranging from 60-105µl[13][14][15]. In practice we observed that it requires around 150µl of whole blood to completely fill the devices and equally dividing that volume in 8 wedges we get approximately 9.3µl serum per wedge. Calculating elution efficiency of Hemaspot HF device, a final eluate dilution of 1:8 in 100µl elution buffer gave us a serum dilution of 1:85 for EIAs. From the above data we could infer that Hemaspot HF gives the highest efficiency of elution as compared to other devices in comparison. This high elution efficiency of Hemaspot HF provides more volume of eluate which can be used for further testing or archiving.

Additives

Since some studies have reported the use of additives such as skim milk, sodium azide and ammonia[16][17][18][19], we experimented with the use of these in our EB to improve the elution efficiency. However, we observed better results for antibody elution against both viruses without the use of these additives. In this study, use of the same elution buffer for both the viruses makes the elution protocol less time-consuming and reduces cost.

**References**

[1] Borremans B. Ammonium improves elution of fixed dried blood spots without affecting immunofluorescence assay quality. Trop Med Int Heal 2014;19:413–6. https://doi.org/10.1111/tmi.12259.

[2] Chanbancherd P, Brown AE, Trichavaroj R, Tienamporn P, Puthakird P, Limpairojn N, et al. Application of dried blood spot specimens for serologic subtyping of human immunodeficiency virus type 1 in Thailand. J Clin Microbiol 1999;37:804–6. https://doi.org/10.1128/jcm.37.3.804-806.1999.

[3] Fenollar F, Raoult D. Diagnosis of rickettsial diseases using samples dried on blotting paper. Clin Diagn Lab Immunol 1999;6:483–8. https://doi.org/10.1128/cdli.6.4.483-488.1999.

[4] Grüner N, Stambouli O, Ross RS. Dried blood spots - Preparing and processing for use in immunoassays and in molecular techniques. J Vis Exp 2015;2015:1–9. https://doi.org/10.3791/52619.

[5] Richardson TC, Chapman D V, Heyderman E. Immunoperoxidase techniques: the deleterious effect of sodium azide on the activity of peroxidase conjugates. J Clin Pathol 1983;36:411–4. https://doi.org/10.1136/jcp.36.4.411.

[6] Saini PK, Webert DW, Judkins JC. Role of Sodium Azide in Reducing Nonspecific Color Development in Enzyme Immunoassays. J Vet Diagnostic Investig 1995;7:509–14. https://doi.org/10.1177/104063879500700415.

[7] Kadjo AF, Stamos BN, Shelor CP, Berg JM, Blount BC, Dasgupta PK. Evaluation of Amount of Blood in Dry Blood Spots: Ring-Disk Electrode Conductometry. Anal Chem 2016;88:6531–7. https://doi.org/10.1021/acs.analchem.6b01280.

[8] Adam BW, Alexander JR, Smith SJ, Chace DH, Loeber JG, Elvers LH, et al. Recoveries of phenylalanine from two sets of dried-blood-spot reference materials: Prediction from hematocrit, spot volume, and paper matrix. Clin. Chem., vol. 46, 2000, p. 126–8. https://doi.org/10.1093/clinchem/46.1.126.

[9] Corran PH, Cook J, Lynch C, Leendertse H, Manjurano A, Griffin J, et al. Dried blood spots as a source of anti-malarial antibodies for epidemiological studies. Malar J 2008;7:1–12. https://doi.org/10.1186/1475-2875-7-195.

[10] Merck. Whatman® protein saver cards 903 Protein saver card (US), pkg of 100 ea | Sigma-Aldrich, n.d.

[11] Spot On Sciences. ELISA Extraction Methods from HemaSpot Standard Curve Preparation, n.d.

[12] Joseph HM, Melrose W. Applicability of the filter paper technique for detection of antifilarial IgG 4 antibodies using the Bm14 filariasis CELISA. J Parasitol Res 2010;2010. https://doi.org/10.1155/2010/594687.

[13] Manak MM, Hack HR, Shutt AL, Danboise BA, Jagodzinski LL, Peel SA. Stability of human immunodeficiency virus serological markers in samples collected as hemaspot and whatman 903 dried blood spots. J Clin Microbiol 2018;56:1–11. https://doi.org/10.1128/JCM.00933-18.

[14] Yamamoto C, Nagashima S, Isomura M, Ko K, Chuon C, Akita T, et al. Evaluation of the efficiency of dried blood spot-based measurement of hepatitis B and hepatitis C virus seromarkers. Sci Rep 2020;10:1–10. https://doi.org/10.1038/s41598-020-60703-1.

[15] Hall JM, Fowler CF, Barrett F, Humphry RW, Van Drimmelen M, MacRury SM. HbA1c determination from HemaSpot^TM^ blood collection devices: comparison of home prepared dried blood spots with standard venous blood analysis. Diabet Med 2019:1–8. https://doi.org/10.1111/dme.14110.

[16] Helfand R, Keyserling H, Williams I, Murray A, Mei J, Moscatiello C, et al. Comparative detection of Measles and Rubella IgM and IgG derived from filter paper blood and plasma samples. J Med Virol 2001;65:751–7. https://doi.org/10.1002/jmv.2100.

[17] Riddell MA, Leydon JA, Catton MG, Kelly HA. Detection of measles virus-specific immunoglobulin M in dried venous blood samples by using a commercial enzyme immunoassay. J Clin Microbiol 2002;40:5–9. https://doi.org/10.1128/JCM.40.1.5-9.2002.

[18] Riddell MA, Byrnes GB, Leydon JA, Kelly HA. Dried venous blood samples for the detection and quantification of measles IgG using a commercial enzyme immunoassay. vol. 81. 2003. https://doi.org/10.1590/S0042-96862003001000004.

[19] Karapanagiotidis T, Riddell M, Kelly H. Detection of rubella immunoglobulin M from dried venous blood spots using a commercial enzyme immunoassay. Diagn Microbiol Infect Dis 2005;53:107–11. https://doi.org/10.1016/j.diagmicrobio.2005.06.017.
