# Supplementary material for: Optimization and Stability Testing of Four Commercially Available Dried Blood Spot Devices for Estimating Measles and Rubella IgG Antibodies
Source: mSphere. 2021 Jul 14;6(4):e00490-21. doi: 10.1128/mSphere.00490-21 (PMC8386427; doi:10.1128/mSphere.00490-21)
Supplement: TABLE S3 [file msphere.00490-21-st003.docx]

|  | **Median coefficient of variation (IQR)** | | **Mean difference, sera – DBS device (SD)** | | | **p-value (paired t-test, sera v. DBS device)** | | **Percent relative difference (sera-DBS)/(sera)** | |
| --- | --- | --- | --- | --- | --- | --- | --- | --- | --- |
| **Device** | **Measles** | **Rubella** | | **Measles** | **Rubella** | **Measles** | **Rubella** | **Measles** | **Rubella** |
| 903 venous | 9.7 (6.3, 11.8) | 8.6 (6.8, 12.8) | | - 18.4 (205.5) | -3.1 (9.7) | 0.76 | 0.29 | -3.7 | -15.9 |
| HF fingerprick | 10.5 (8, 13.7) | 10.9 (7.2, 14.9) | | 72.2 (120.0) | 1.6 (5.0) | 0.06 | 0.28 | 3.6 | -0.5 |
| HF venous | 9 (7.2, 12.8) | 14.6 (9.6, 17.1) | | 31.4 (150.5) | -2.3 (5.7) | 0.48 | 0.18 | -1.3 | -5.7 |
| TropBio venous | 8.8 (6.2, 13.2) | 14.5 (10.2, 22.2) | | 67.5 (272.0) | -1.8 (12.0) | 0.41 | 0.62 | 4.0 | -12.0 |
