# Supplementary material for: Optimization and Stability Testing of Four Commercially Available Dried Blood Spot Devices for Estimating Measles and Rubella IgG Antibodies
Source: mSphere. 2021 Jul 14;6(4):e00490-21. doi: 10.1128/mSphere.00490-21 (PMC8386427; doi:10.1128/mSphere.00490-21)
Supplement: TABLE S4 [file msphere.00490-21-st004.docx]

| **Storage temperature and duration** | **Measles, percent of baseline (%)** | | | **Rubella, percent of baseline (%)** | | |
| --- | --- | --- | --- | --- | --- | --- |
|  | **4^°^C (cold room)** | **Ambient temperature** | **45^°^C (incubator)** | **4^°^C (cold room)** | **Ambient temperature** | **45^°^C (incubator)** |
| **Day 7** | 102 | 97 | 68 | 101 | 97 | 57 |
| **Day 15** | 101 | 95 | 43 | 95 | 94 | 32 |
| **Day 30** | 100 | 92 | 30 | 91 | 89 | 20 |
| **Day 60** | 98 | 87 | 23 | 87 | 83 | 13 |
| **Day 90** | 96 | 77 | 18 | 93 | 73 | 11 |
