# Supplementary figures and images for: Optimization and Stability Testing of Four Commercially Available Dried Blood Spot Devices for Estimating Measles and Rubella IgG Antibodies
Source: mSphere. 2021 Jul 14;6(4):e00490-21. doi: 10.1128/mSphere.00490-21 (PMC8386427; doi:10.1128/mSphere.00490-21)

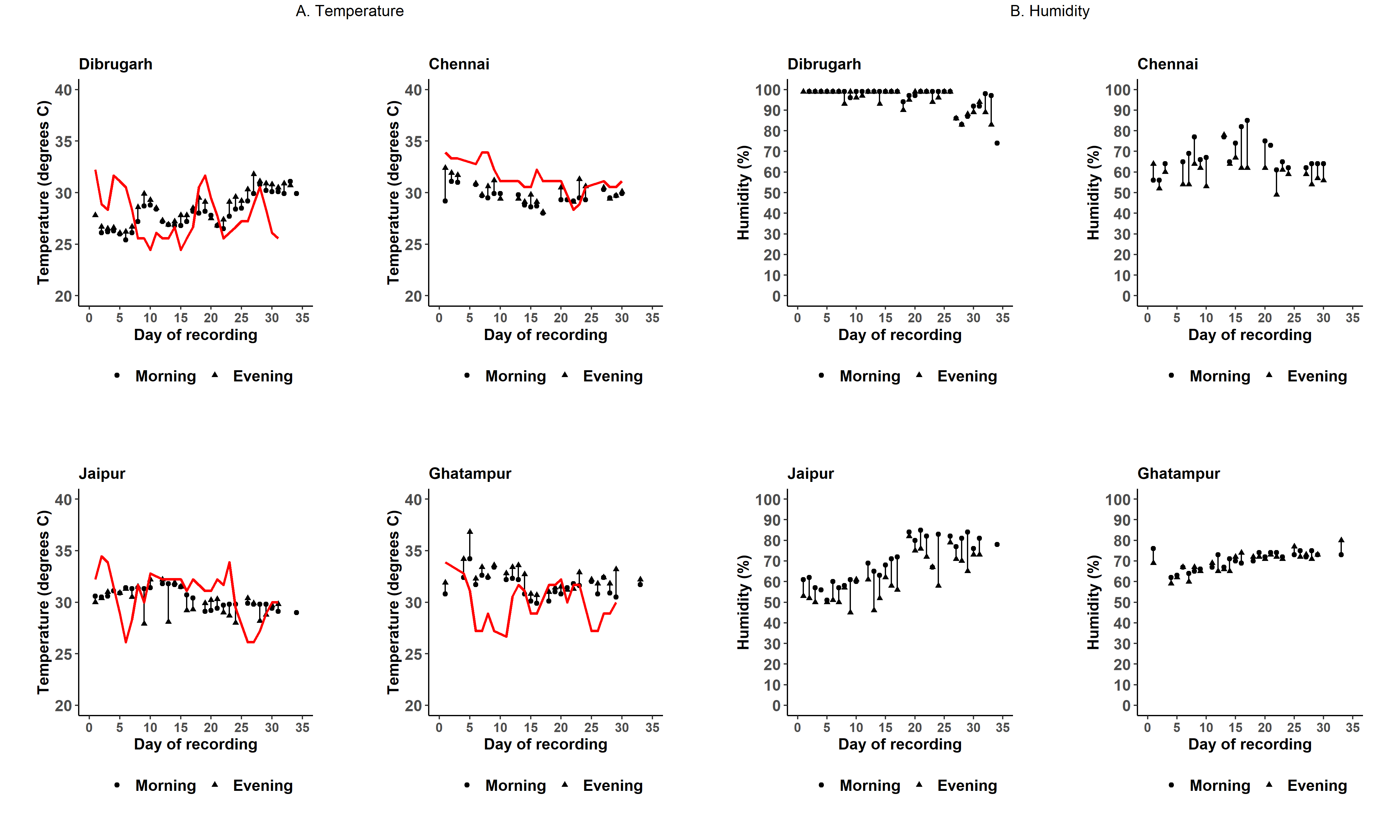

Supplement: FIG S1 [file msphere.00490-21-sf001.tif]
