# Supplementary material for: Optimization and Stability Testing of Four Commercially Available Dried Blood Spot Devices for Estimating Measles and Rubella IgG Antibodies
Source: mSphere. 2021 Jul 14;6(4):e00490-21. doi: 10.1128/mSphere.00490-21 (PMC8386427; doi:10.1128/mSphere.00490-21)
Supplement: TABLE S1 [file msphere.00490-21-st001.docx]

|  | Positive | Equivocal | Negative |
| --- | --- | --- | --- |
| Measles (mIU/mL) | > 275 | > 200 - < 275 | < 200 |
| Rubella (IU/mL) | > 11 | > 8 - < 11 | < 8 |
