# Supplementary material for: Optimization and Stability Testing of Four Commercially Available Dried Blood Spot Devices for Estimating Measles and Rubella IgG Antibodies
Source: mSphere. 2021 Jul 14;6(4):e00490-21. doi: 10.1128/mSphere.00490-21 (PMC8386427; doi:10.1128/mSphere.00490-21)
Supplement: TABLE S2 [file msphere.00490-21-st002.docx]

| **Plan** | **Storage location** | **Storage condition** | **Timepoint (Day)^a^** | | | | | | |  |
| --- | --- | --- | --- | --- | --- | --- | --- | --- | --- | --- |
|  |  |  | **0** | **7** | **15** | **30** | **60** | **90** | |  |
| Laboratory setting  (July 2019)^a^ | Pune, Maharashtra | Serum -20 ° C | ✓ |  |  |  |  |  | |  |
|  |  | **DBS (HemaSpot HF Devices)** | | | | | | |  | |
|  |  | 4° C | ✓^b^ | ✓ | ✓ | ✓ | ✓ | ✓ | |  |
|  |  | Ambient temperature  (22° C – 25° C) |  | ✓ | ✓ | ✓ | ✓ | ✓ | |  |
|  |  | 45° C |  | ✓ | ✓ | ✓ | ✓ | ✓ | |  |
| Field settings (July 2019) | Dibrugarh, Assam (foothills), ICMR-Regional Medical Research Centre | Moderate Temperature, High Humidity |  |  |  |  |  |  | |  |
|  | Chennai, Tamil Nadu (Coastal region), ICMR- National Institute of Epidemiology | Moderate Temperature, Moderate Humidity |  |  |  | ✓ |  |  | |  |
|  | Jaipur, Rajasthan (Desert), Sawai Man Singh Medical College | High Temperature, Moderate Humidity |  |  |  |  |  |  | |  |
|  | Ghatampur, Uttar Pradesh (northern plains),ICMR- Model Rural Health Research Unit | High Temperature, High Humidity |  |  |  | ✓ |  |  | |  |
